# The Boundary Conditions of High-Performance Work Systems–Organizational Citizenship Behavior Relationship: A Multiple-Perspective Exploration in the Chinese Context

**DOI:** 10.3389/fpsyg.2021.743457

**Published:** 2022-01-20

**Authors:** Bo Zhang, Lihua Liu, Fang Lee Cooke, Peng Zhou, Xiangdong Sun, Songbo Zhang, Bo Sun, Yang Bai

**Affiliations:** ^1^College of Economics and Management, Beijing University of Chemical Technology, Beijing, China; ^2^Department of Management Consulting, Beijing HIWING Scientific and Technological Information Institute, Beijing, China; ^3^Monash Business School, Monash University, Melbourne, VIC, Australia; ^4^Cardiff Business School, College of Arts, Humanities and Social Sciences, Cardiff University, Cardiff, United Kingdom; ^5^College of Business Administration, Capital University of Economics and Business, Beijing, China; ^6^Department of Surgery, PLA Rocket Force Characteristic Medical Center, Beijing, China

**Keywords:** high-performance work systems, boundary effect, organizational identification, error aversion culture, organizational citizenship behavior

## Abstract

This research synthesizes social exchange, organizational culture, and social identity theories to explore the boundary conditions of the relationship between high-performance work systems and employee organizational citizenship behavior. In particular, it draws on the China-specific management context. In this country, in spite of the wide use of a long-term-oriented and loose-control-focused Western-styled strategic human resource management (HRM) model, a short-term-focused and tight-control-oriented error aversion culture is still popular. The study uses multi-source individual-level survey data in a large state-owned enterprise to test the hypotheses. It is found that employee-experienced, Western-styled high-performance work systems positively impact a China-specific employee’s organizational citizenship behavior (OCB), and the—joint—moderation effects of employee-perceived error aversion culture and organizational identification are significant. The research findings deepen the understanding of the HRM-OCB relationship by demonstrating that culture and identity can jointly adjust the effects of HRM on OCB. The findings also challenge an established argument in the HRM-OCB literature that compatibility between employees’ personalities and organizational values – organizational identification – can enhance OCB.

## Introduction

Organizational citizenship behavior (OCB) refers to “individual behavior that is discretionary, not directly or explicitly recognized by the formal reward system, and in the aggregate promotes the efficient and effective functioning of the organization” (Organ et al., 2006, p. 3). Employees’ OCB is very important for firms to generate competitive advantage in a dynamic business environment (Organ et al., 2006; Podsakoff et al., 2009), such as China (Sun et al., 2007; Zhang et al., 2014; Newman et al., 2015). Strategic human resource management (HRM) scholars have been increasingly interested in investigating the HRM-OCB relationship in Western countries (Snape and Redman, 2010; Halbesleben et al., 2014; Caniëls and Hatak, 2019; Valeau and Paillé, 2019; Asplund, 2020; Hernaus et al., 2021) and in China (Sun et al., 2007; Gong et al., 2009, 2010; Shen and Benson, 2014; Zhang et al., 2014). Most of the studies have reported positive impacts of HRM systems or practices on OCB through social exchange mechanisms.

However, HRM-OCB relationship research still suffers from both theoretical and methodological shortcomings. Theoretically, there is an overreliance on the use of social exchange theory and overlooking boundary conditions when studying the HRM-OCB relationship (Zellars and Tepper, 2003; Snape and Redman, 2010). These two theoretical limitations are interrelated, and call for the exploration of the boundary effects of HRM-OCB relationship from multiple theoretical perspectives. Therefore, our main purpose is to explore the HRM-OCB relationship from an interactionist approach of three frequently used perspectives in HRM performance and employee behavior literature, including social exchange, organizational culture, and social identity theories.

According to social exchange theory, HRM systems can induce employee OCB through shaping the employee–organization social exchange relationship (Tsui et al., 1997; Gong et al., 2010). HRM scholars often use high-performance work systems (HPWS), “an ensemble of HR practices that aims at getting more from workers by giving more to them” (Baron and Kreps, 2003, p. 189), to investigate HRM-OCB linkages. The implementation of HPWS can provide employees with social resources, such as prestige, trust, organizational support, skill development, and career advancement. In return, employees reciprocate organization with OCB (Snape and Redman, 2010). Therefore, we propose and test HPWS’ impacts on employee OCB.

Organizational culture refers to a set of shared mental models that reflect group life, that is, the way we perceive the world, solve problems, react emotionally to what we perceive and how we value things (Schein and Schein, 2016). HRM systems’ effects on employee outcomes are varied across different organizational cultures because of the different degrees to which organization’s HRM systems are consistent with organizational cultures (Jackson et al., 2014; Cooke, 2017). The dominant approach in China-based literature is to explore organizational cultures’ role in strengthening HRM systems’ effectiveness (e.g., Xiao and Tsui, 2007; Wei et al., 2008; Tsui et al., 2010). However, certain China-specific organizational cultures might offset the effectiveness of Western-styled HRM systems (Zhang and Jia, 2010), for example, an error aversion culture. Unfortunately, no research has investigated the boundary effects of an error aversion culture in HRM-OCB research. Thus, we propose and investigate the moderating role of an error aversion culture in HPWS-employee OCB relationship.

Social identity theory, particularly the construct of organizational identification, is also frequently used in the HRM field (Alvesson and Kärreman, 2007), as well as in OCB research (Tavares et al., 2016). Organizational identification refers to the “cognition of membership of a group and the value and emotional significance attached to this membership” (Tajfel, 1978, p. 63). Whether an employee holds a self-identity or organizational identity may shift an employee’s foci (self versus organization) of behavioral decision in socially exchanging with the organization (Tavares et al., 2016). “Strategic HRM scholars typically acknowledge that HRM systems cannot be fully understood without considering their interrelationships with other elements of an organization to which it is inextricably bound” (Jackson et al., 2014, p. 4). Therefore, we propose and test the three-way interactive effects on employee OCB of HPWS, error aversion culture, and organizational identification.

Methodologically, this study raises two concerns to further enhance the validity of empirical investigations, namely the type of OCB and the measurement of HRM systems and organizational culture. In terms of the former, previous China-based HRM-OCB studies often measured employee OCB by using scales developed in the Western context, whereas Chinese workers might have different patterns of OCB (Farh et al., 2004). To capture the characteristics of the China-specific management context, we focus on a China-specific employee OCB. As for the latter, “employees may perceive or experience differences in exposure to work practices” (Liao et al., 2009, p. 274). The experience-based, rather than observation-based or description-based, measurement of HRM practices have a stronger relationship with employee’s outcomes (Wang et al., 2020). Organizational culture scholars suggest that employee behavioral choices are constrained by organizational culture through employee perception (Zou et al., 2009). Thus, we measure HPWS by using employee-experienced HPWS and measure error aversion culture by using employee-perceived error aversion culture.

This research strives for building up a more complicate picture of HRM-OCB’s linking mechanism. Integrating the three theories to study HRM-OCB relationship can contribute to the literature by putting HRM systems back into a more complex but more real context. Specifically, the norm of social exchange relationships may be a universally accepted principle (Gouldner, 1960; Blau, 1986), yet the degree to which people and cultures apply social exchange relationship principles varies (Cropanzano and Mitchell, 2005).

## Literature Review and Hypothesis Development

### Situating HPWS-OCB Research in the Chinese Cultural Context

This study investigates how a Western-developed management tool influences China-specific employee behavior in an organizational culture with Chinese characteristics. Firstly, a strategic HRM view was widely accepted in Chinese firms, which has led to the use of high-performance work practices or systems (Kim et al., 2012; Xiao and Cooke, 2020). High-performance work practices or systems have been reported as impacting on a series of work outcomes (Kim et al., 2012), including OCB (Sun et al., 2007; Shen and Benson, 2014; Zhang et al., 2014).

Second, like HPWS, OCB was originally developed in Western context, which was argued to be more or less deviated from citizen behavior in Chinese organization. Based on previous research in OCB’s structure in Western context and investigations in Chinese organizations, Farh et al. (1997) developed five-dimension construct of OCB to capture Chinese worker’s citizenship behaviors in Chinese organization. Two of them – protecting company resources and interpersonal harmony – did not appear in the Western scale, which “can be attributed to their [Chinese] cultural roots” (Farh et al., 1997, p. 428). The dimension of protecting company resources has its cultural origins in Chinese familistic collectivism (Farh et al., 1997). The cultural root of interpersonal harmony dimension of OCB is

A cherished cultural value of interpersonal harmony found in Chinese societies … it is a common practice in traditional Chinese societies for anyone who first violates interpersonal harmony, for whatever reason, to take a much larger share of the blame, no matter whether his or her behavior is justifiable (Farh et al., 1997, p. 430).

We focus on the interpersonal harmony dimension of OCB, not only because it is strongly emphasized in Chinese culture (Yang, 1993), but also because it is regulated by culture where employees perform it without using their personal discretion. Although the dimension of protecting company resources is also a valid OCB form in China, it may be managed by a firm’s regulations and laws. In such a case, protecting company resources it will not be a behavior in association with personal discretion.

Secondly, HRM scholars have increasingly called for academic attention to placing HRM in context (Jackson et al., 2014; Kaufman, 2015; Cooke, 2017) when exploring how HRM systems function. Organizational culture is emphasized as a crucial element of organizational context that intervenes in the effectiveness of HRM systems (Jackson et al., 2014). Although the term “error aversion culture” was coined in a Western management context (van Dyck et al., 2005; van Dyck, 2009), it is prevalent in the Chinese management context. In Chinese firms, an error aversion culture has been found to inhibit employer-expected employee outcomes, such as innovation, decreased turnover rate (Du and Chen, 2019), and employee voice. This culture also abated the positive effects of employee personal traits on firm-expected work outcomes (Ma and Liu, 2019).

**Figure 1:**
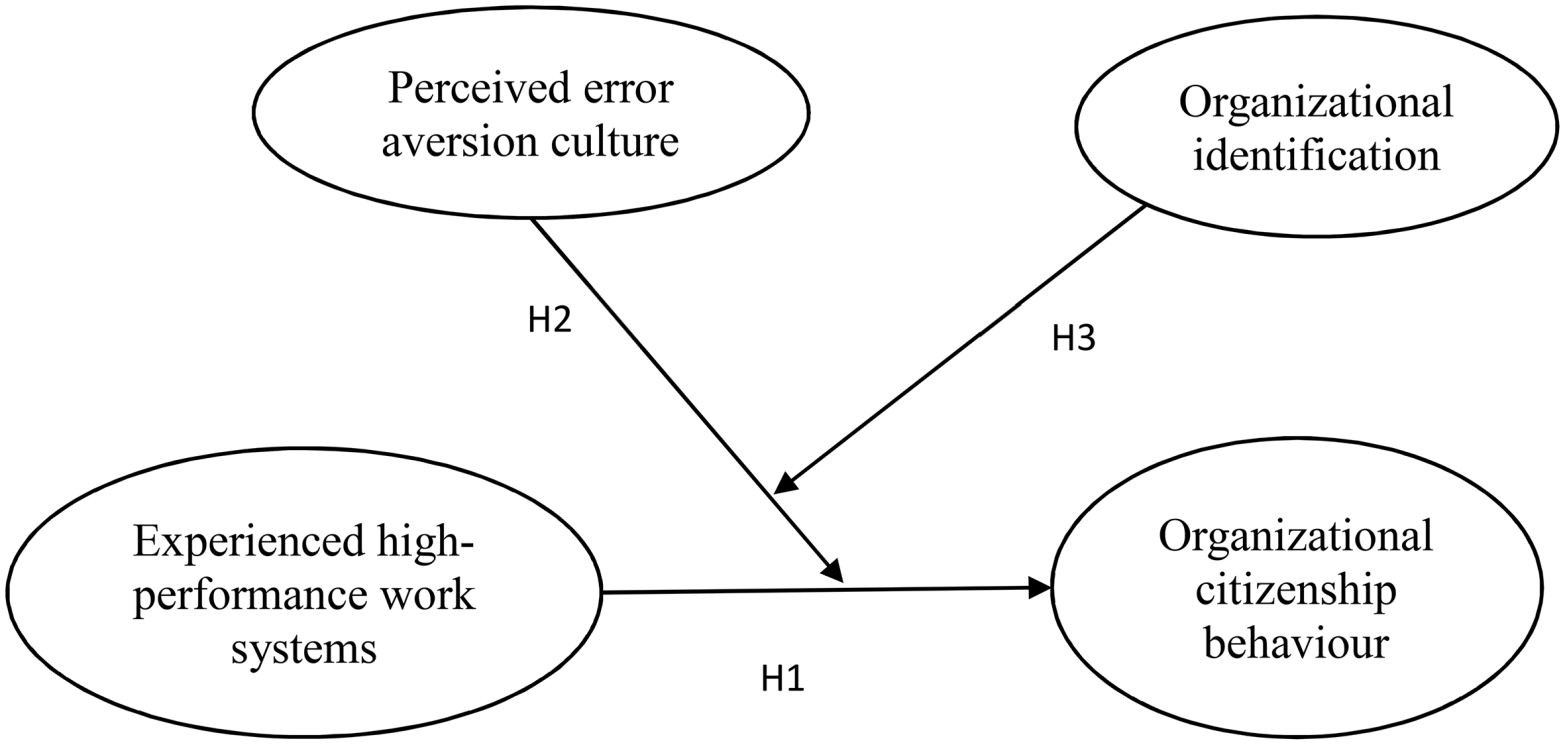
Conceptual model.

Finally, there is a tradition of collectivism among employees, which emphasizes the value of wholeness and the concern of collective interests. It has been argued that employees identifying with the organization’s values and norms will exhibit more OCB (e.g., Newman et al., 2015; Callea et al., 2016; Shen et al., 2018). However, we challenge this view, because identifying with an error aversion culture will strengthen the offsetting effects between the error aversion culture and HPWS on employee OCB. Thus, it is worth exploring the interactive effects on a China-specific employee’s OCB of a Western-styled HRM system (e.g., HPWS), an error aversion culture, and employee organizational identification. The conceptual model in this research is shown in Figure 1.

### Employee-Experienced HPWS and OCB

Social exchange theory focuses on the resources that people obtain from, and contribute to, social interactions (Blau, 1986; Molm et al., 2001). Exchange parties follow the principles of reciprocity (i.e., the recipient is obligated to return a benefit to the party who furnishes such a benefit) and equivalence (i.e., the recipient returns benefits of equivalent value: Gouldner, 1960). HRM systems can shape the nature of the employee–organization social exchange relationship (Tsui et al., 1997) to induce employee OCB (Gong et al., 2009, 2010; Snape and Redman, 2010). HRM systems offer social resources, as well as economic resources, to employees. Such a function of HRM systems can be underpinned by a sensemaking process “whereby organizational members translate an organizational event and construct a meaningful explanation for that event” (Greenberg, 1995, p. 185). For example, performance-based pay can represent organization investment in economic resources, such as time and money, in employees’ performing at work. Through sensemaking, employees can also interpret the use of such time and money as an expectation of better performance or strategy-oriented contribution. Expectation here is a social resource. Based on the principles of reciprocity and equivalence (Gouldner, 1960), employees feel obliged to reciprocate their organizations with their social resources according to the social resources they have received from them. Performance-based pay can lead to expectations (social resources) that besides time and money (economic resources), employees receiving the organization’s expectation will feel obliged to generate great effort in pursuit of performance goals. Therefore, there may be a positive relationship between HPWS and employee OCB.

Although there is no consensus on the elements of HPWS, it usually embraces practices such as extensive selection for job applicants, participation, extensive training, employee ownership programs, results-based performance appraisal, team-based appraisal, profit-sharing practices, etc. (Lepak et al., 2006; Jiang et al., 2012). Through extensive selection for job applicants, employees selected are likely to feel that the firm recognizes their personal traits, and values their knowledge skills and abilities. Participation practices and comparatively high pay may be seen as recognition of their value and importance to the organization (Gong et al., 2009). Hence, they may experience enhanced self-importance and self-image (Blau, 1986; Gong et al., 2010). Extensive training represents the organization’s investment in trained employees’ skill development (Takeuchi et al., 2007). Career development means the organization’s care for and commitment to employees’ futures in the firm. Employee ownership programs also deliver the sense that a firm depends on its employees for its development. When implementing such practices, an organization’s trust is likely to be perceived by employees (Arthur, 1994; Gong et al., 2010).

Many HPWS practices, such as results-based performance appraisal and employee participation practices, are not limited to specific or current jobs. Such practices may grant an employee autonomy or loose control and job influence (Snape and Redman, 2010). Through participation practices, the organization offers opportunities for employees to hold shared objectives and to cooperate with co-workers for achieving them (Foss et al., 2014). Working in such circumstances, employees are likely to develop trust with each other. Team-based appraisal and profit-sharing practices deliver shared responsibilities among co-workers for work outcomes and their potential rewards.

As a system, these high-performance work practices offer multiple social resources (e.g., self-image, trust, empowerment or loose control, and shared goals and responsibilities among co-workers), and the multiplicity of systems of HR practices sets and reinforces the tone of the social exchange relationship with employees (Gong et al., 2010). Employees are likely to perceive self-image, trust, empowerment, shared goals and responsibilities among co-workers. They are then likely to reciprocate by expanding their definition of job responsibilities and engage in more OCB (Snape and Redman, 2010), such as building harmony with co-workers (Farh et al., 1997). Therefore, we propose that:

*Hypothesis 1:* Employee-experienced HPWS will be positively related to OCB.

### The Role of Perceived Error Aversion Culture

This culture means that organizations either pay little attention to errors or hold the view that errors should or could be fully eliminated (van Dyck et al., 2005). In Chinese firms, there coexist HPWS and an error aversion culture. The former is a long-term-oriented and loose-c model (Gong et al., 2010), whereas the latter is a short-term- and tight-control-oriented approach in organization (van Dyck, 2009). The two contradictory management elements may offset each other’s effects (Zhang and Jia, 2010).

The relationship between employee-experienced HPWS and employee OCB may be weakened when employees perceive an error aversion culture for at least two reasons. On the one hand, social resources provided by the employee-organization social exchange relationship and shaped by HPWS are likely to be damaged or offset by social resources generated by an error aversion culture. Specially, in an error aversion culture, individuals who make errors are not accepted by others, or indeed are blamed or punished. These cultural experiences damage their maker’s self-image (Hockey and Sauer, 1996). In addition, people tend to avoid talking about errors that occurred, and error makers may cover up their errors (van Dyck, 2009). Such interactions among co-workers will harm the trust perceived by employees. Finally, firms strive to prevent the occurrence of errors in an error aversion culture (van Dyck et al., 2005), which leads them to put their employees under tight surveillance and control. Employees working in such a situation will feel distrust from the organization and feel they are losing control over their job. Hence, the overall quality of the resources offered by the employee–organization social exchange relationship shaped by HPWS are reduced, resulting in a lower level of likelihood for employees to reciprocate the expected OCB (also see Zhang and Jia, 2010).

On the other hand, employees are willing to respond to organizational practices that meet their needs and are in their favor (Nishii and Wright, 2008). In an error aversion culture, avoidance of errors and punishment is in individual employees’ favor. To protect their self-interest and avoid making mistakes, employees will reluctantly reciprocate OCB through the social exchanges shaped by HPWS that induce them to do so. Hence, we propose that:

*Hypothesis 2:* The relationship between employee-experienced HPWS and OCB will be moderated by a perceived error aversion culture, such that the relationship will be stronger when the perceived error aversion culture is low.

### The Role of Organizational Identification

SIT assumes that individuals tend to have a positive self-concept and that their identity is partly based on membership in social groups (Gautam et al., 2004). Social identities play a crucial role for employees’ attitudes and behaviors in organizations because being a member of an organizational grouping helps to answer the question of “Who am I?” (Ashforth and Mael, 1989). The organization serves as a potentially social category, where employee’s needs of belonging and security can be satisfied (Hogg and Terry, 2000). Organizational identification may strengthen the moderating effects of error aversion culture on the relationship between employee-experienced HPWS and employee OCB. According to the literature, whether or not an employee holds a strong self-identity or organizational identity may shift an employee’s foci (self versus organization) of behavioral decision in exchanges with the organization (van Knippenberg and Sleebos, 2006; van Knippenberg and Schippers, 2007; Tavares et al., 2016; Wikhamn et al., 2021). In the employee-organization social exchanges shaped by HPWS, the organization provides social resources to individual employees idiosyncratically, which means that such exchanges hinge on an employee’s self-focused discretion (van Knippenberg and Schippers, 2007). Conversely, organizational culture in itself is the shared values among employees. It generates constraints to align individual employees’ behaviors with the organization. In this sense, it fosters a sense of collective or organizational focus.

Organizational identification is concerned with the perception of “oneness” with the organization (Mael and Ashforth, 1992). Employees with a high level of organizational identification not only perceive shared interests with the organization, but also feel proud of belonging to and being acknowledged in the organization (Mael and Ashforth, 1992; Smidts et al., 2000). That said, with high organizational identification, employees identify with the organization’s values, accept the organization’s norms, and cherish recognition in the organization. Consequently, they will experience and reciprocate behaviors for the social resources received from the error aversion culture more strongly than those from the individual idiosyncratic social exchanges shaped by HPWS. Thus, we propose that:

*Hypothesis 3:* Perceived error aversion culture, and organizational identification will jointly moderate the relationship between employee-experienced HPWS and OCB, such that the relationship will be the strongest when perceived error aversion culture and organizational identification are both low.

## Materials and Methods

### Sample and Data Collection

Survey data were collected in June 2018 using questionnaires to a large state-owned enterprise in China. Because the scales employed were developed in English, we used a translation and back-translation procedure to guarantee the validity of the scale (Brislin, 1970). Two questionnaires were prepared. The first was completed by employees and contains questions about employee personal details, experienced HPWS, and perceived error aversion culture. The second contains questions about employee OCB and was completed by their immediate supervisor.

We conducted a pilot study of 22 employees and five HR managers before the main survey. Based on the feedback from the pilot study, we refined the wording of a few questions. Then, the questionnaires were given to 20 employees and five HR managers who had not participated in the pilot study: the respondents understood the questions appropriately.

Two thousand pairs of questionnaires were sent to potential respondents in 2018. After 3 weeks, 1,667 pairs were returned, which was a response rate of 83.35%. After removing questionnaires of low response quality, a total of 1,231 pairs of valid questionnaires remained. The descriptive analysis results are presented in Table 1.

**Table 1:** Sample characteristics.

Demographic characteristic	Category	Percentage (%)	Demographic characteristic	Category	Percentage (%)
Gender	Male	68.9	Organization tenure (year)	5 and shorter	43.4
	Female	31.1		6–10	23.3
Career path	Managerial staff	40.9		11–15	12.3
	Technical staff	54.6		16–20	6.2
	Supporting staff	4.5		21–25	7.8
Age (year)	30 and younger	29.3		Longer than 25	7.0
	31–35	27.4	Education	High school	0.2
	36–40	17.1		Professional school	0.5
	41–45	12.3		Undergraduate	72.5
	46–50	9.2		Master degree	23.0
	51–55	4.0		PhD	3.4
	56 & above	0.7		Others	0.3

Responses of 149 managers and 1,231 employees were used for data analysis; the personal characteristics reported in the table are only for employees and do not include the managers who rated employee’s organizational citizenship behavior.

### Measures

All the items in the scales used in this study were rated on a Likert scale that ranged from 1 (completely disagree) to 7 (completely agree).

#### Employee-Experienced High-Performance Work Systems

Employee-experienced HPWS was measured by 19 items selected and adapted from Delery and Doty (1996). Employees were asked to evaluate their own experienced high-performance work practices. A sample item was “I regularly receive extensive training programs are provided for individuals in this job.” The Cronbach’s alpha value was 0.942.

#### Perceived Error Aversion Culture

Four items were selected and adapted from van Dyck et al. (2005). Participants were asked to rate their own perception about dealing with errors in the organization. A sample item was “people are often afraid of making errors.” As van Dyck et al. (2005) found, this construct is unidimensional and the Cronbach’s alpha value is 0.904.

#### Organizational Identification

Four items were adopted from Smidts et al. (2000). A sample item was “I feel strong ties with my company’. Respondents were asked to answer “to what extent do you (dis)agree with the following descriptions.” As with Smidts et al.’s (2000) finding, this construct is unidimensional. The Cronbach’s alpha value is 0.935.

#### Organizational Citizenship Behavior

We employed interpersonal harmony OCB in Farh et al.’s (1997) scale, which was measured by 4 items. A sample item was “She or he uses illicit tactics to seek personal influence and gain with harmful effect on interpersonal harmony in the organization.” Respondents were asked to rate “to what extent do you agree with the description about the behaviors of this employee.” The Cronbach’s alpha value is 0.863.

#### Control Variables

We included a series of control variables in our analysis. The control variables included gender (male as 1, female as 0), age, education background (undergraduate as 1, the rest as 0), career path (managerial staff as 1, the reset as 0), and organization tenure. We controlled the effects of these personal or job-related variables because they can affect employees’ behaviors at work. Moreover, these variables were also employed as control variables in previous studies when investigating antecedents of OCB (e.g., Farh et al., 1997; Bergeron et al., 2012; Shen and Benson, 2014).

## Data Analysis

### Factor Analysis

We conducted confirmatory factor analysis using MOS23. The results in Table 2 show that a four-factor model is better [χ^2^(125) = 372.392, CFI = 0.984, TLI = 0.981, RMSEA = 0.040, RMR = 0.066] than others.

**Table 2:** Confirmatory factor analysis.

Model	χ^2^	df	χ^2^/df	CFI	TLI	RMSEA	RMR
Single-factor model (EEHPWS + PEAC+OI + OCB)	6520.435	131	49.774	0.597	0.530	0.199	0.286
Two-factor model (EEHPWS + PEAC, OI + OCB)	4482.300	130	34.479	0.726	0.677	0.165	0.264
Three-factor model (EEHPWS, PEAC, OI + OCB)	1596.586	128	12.473	0.907	0.889	0.097	0.090
Four-factor model (EEHPWS, PEAC, OI, OCB)	372.392	125	2.979	0.984	0.981	0.040	0.066
Four Factor + CMV	360.984	124	2.911	0.985	0.982	0.039	0.062

EEHPWS: employee-experienced high-performance work systems; PEAC: perceived error aversion culture; OI: organizational identification; OCB: organizational citizenship behavior.

### Hypothesis Testing

Table 3 shows the mean value, standard deviation, and correlation coefficient values of variables used.

**Table 3:** Mean, standard deviation, and correlation.

Variables	Mean	S.D.	Cronbach’s alpha score	1	2	3	4	5	6	7	8
Gender	---	---	---								
Age	2.595	1.500	---	0.087^**^							
Education	---	---	---	−0.028	−0.121^**^						
Career path	---	---	---	−0.026	−0.086^**^	−0.028					
Job tenure	2.351	1.650	---	0.063^*^	0.735^**^	−0.129^**^	−0.061^*^				
Experienced high-performance work systems	4.711	1.182	0.942	0.009	−0.136^**^	−0.224^**^	−0.052	−0.187^**^			
Perceived error aversion culture	3.112	1.327	0.904	−0.073^*^	0.081^**^	0.074^**^	0.003	0.099^**^	−0.318^**^		
Organizational identification	5.658	1.334	0.935	0.011	0.031	−0.096^**^	−0.070^*^	−0.053^*^	0.589^**^	−0.271^**^	
Organizational citizenship behavior	6.414	0.751	0.863	0.051	0.057^*^	−0.162^**^	−0.084^*^	−0.008	0.453^**^	−0.246^**^	0.521^**^

Responses of 149 managers and 1,231 employees were used for data analysis; the personal characteristics employed in the table are only for employees and do not include the managers who rated employee’s organizational citizenship behavior; S.D: standard deviation.

** *p* < 0.01;

* *p* < 0.05.

All the variables were centered to reduce non-essential multicollinearity (Aiken and West, 1991). To test the hypotheses, we used SPSS 22 to conduct hierarchical regressions. The results are shown in Table 4. Hypothesis 1 states that employee-experienced HPWS will be positively related to OCB. In Model 2, the result shows that employee-experienced HPWS is positively and significantly related to OCB (β = 0.202, *p* < 0.001). Thus, Hypothesis 1 is supported.

**Table 4:** Effects of HPWS on OCB and the moderating role of perceived error aversion culture and organizational identification.

	Organizational citizenship behavior			
	M1	M2	M3	M4
	Standardized β	Standardized β	Standardized β	Standardized β
**Control variable**				
Gender	0.041	0.034	0.034	0.035
Age	0.116^**^	0.070^*^	0.074^*^	0.073^*^
Education	−0.165^***^	−0.069^***^	−0.068^**^	−0.069^**^
Career path	−0.085^**^	−0.043	−0.039	−0.011
Job tenure	−0.123^**^	−0.008	−0.010	−0.007
**Main effect**				
Employee-experienced HPWS	XX	0.202^***^	0.214^***^	0.222^***^
Perceived Error Aversion Culture	XX	−0.079^**^	−0.078^**^	−0.149^***^
Organizational Identification	XX	0.368^***^	0.353^***^	0.365^***^
**Two-way Interaction**				
Employee-experienced HPWS X perceived error aversion culture	XX	XX	−0.089^**^	−0.102^***^
Employee-experienced HPWS X organizational identification	XX	XX	−0.019	−0.036
Perceived error aversion culture X organizational identification	XX	XX	0.042	0.127^***^
**Three-way Interaction**				
Employee-experienced HPWS X perceived error aversion culture X organizational identification	XX	XX	XX	0.164^***^
VIF value	1.008–2.196	1.015–2.239	1.020–2.243	1.020–2.320
F value	11.172^***^	72.994^***^	54.166^***^	52.541^***^
R^2^	0.044	0.323	0.328	0.341
△R^2^	0.044^***^	0.280^***^	0.005^*^	0.013^***^

Responses of 149 managers and 1,231 employees were used for data analysis; the personal characteristics employed in the table are only for employees and do not include the managers who rated employee’s organizational citizenship behavior.

* *p* < 0.05;

** *p* < 0.01;

*** *p* < 0.001.

**Figure 2:**
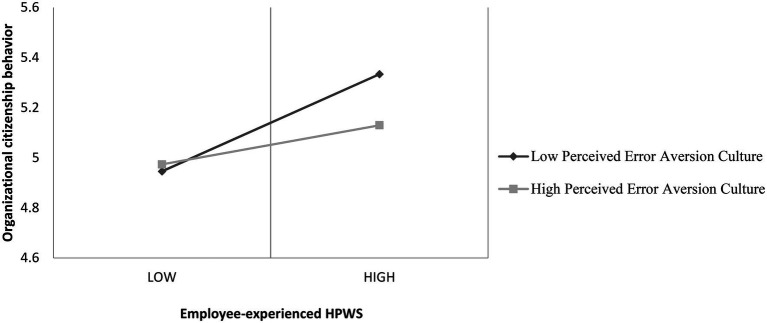
The moderating effects of perceived error aversion culture on the HPWS-OCB relationship.

Hypothesis 2 states that the relationship between HPWS and OCB will be moderated by perceived error aversion culture, such that the relationship will be stronger when perceived error aversion culture is low. Model 3 in Table 4 shows that the interactive term of employee-experienced HPWS is significantly and negatively related to OCB (β = −0.089, *p* < 0.01). Figure 2 shows that with low perceived error aversion culture (1 s.d. above the mean), HPWS is more positively associated OCB than with high perceived error aversion culture (1 s.d. below the mean). We also conducted a slope test and slope difference tests (Dawson and Richter, 2006), and the results (see Table 5 for details) supported Hypothesis 2. Therefore, Hypothesis 2 is supported.

**Table 5:** Simple slops test two-way and three-way interactions.

Pairs of comparisons	Slope	Boot SE	T test	Bootstrap 95%	
				Boot LLCI	Boot ULCI
Low perceived error aversion culture	0.186	0.028	6.681^***^	0.131	0.241
High perceived error aversion culture	0.085	0.025	3.405^***^	0.036	0.133
Low organizational identification, low perceived error aversion culture	0.281	0.037	7.674^***^	0.209	0.352
Low organizational identification, high perceived error aversion culture	0.038	0.031	1.244	−0.022	0.099
High organizational identification, low perceived error aversion culture	0.120	0.033	3.692^***^	0.056	0.184
High organizational identification, high perceived error aversion culture	0.124	0.031	4.059^***^	0.065	0.185

Low = mean − 1 standard deviation; High = mean + 1 standard deviation.

*** *p* < 0.001.

**Figure 3:**
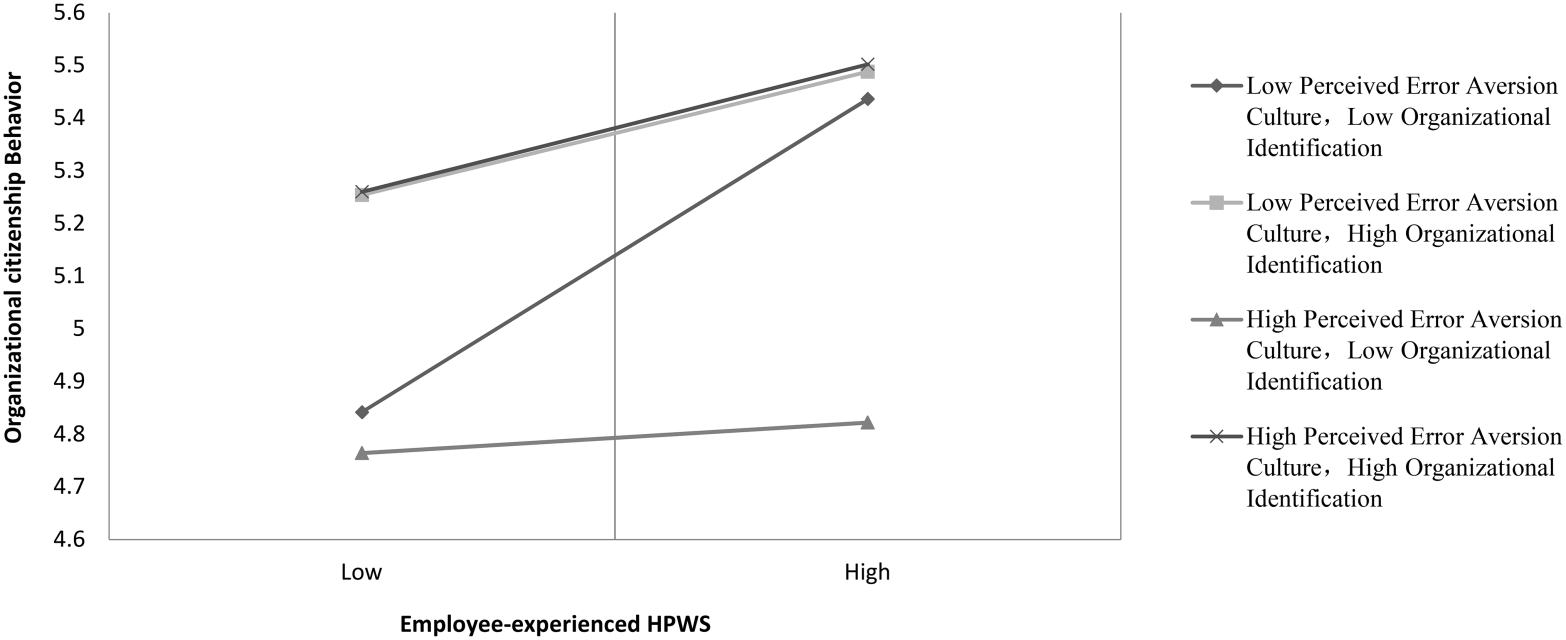
The three-way interactive effects of HPWS, perceived error aversion culture, and organizational identification on OCB.

Hypothesis 3 states that the moderating effects of perceived error aversion culture on the relationship between employee-experienced HPWS and OCB will be augmented by organizational identification, such that the relationship will be the strongest when perceived error aversion culture and organizational identification are both high. In Model 4, the three-way interactive term of employee-experienced HPWS, perceived error aversion culture, and organizational identification is significantly related to OCB (β = 0.164, *p* < 0.001). We plotted the three-way interaction in Figure 3. The simple slopes and slope difference tests are given in Table 5. As shown in Figure 3 and Table 5, Hypothesis 3 is supported.

## Discussion

The overall purpose of this study is to investigate the boundary effects of perceived error aversion culture and organizational identification on the relationship between employee-experienced HPWS and OCB. Findings of the study support our conceptualization and have theoretical and empirical implications.

### Theoretical Implications

Firstly, our conceptualization, based on social exchange theory and supported by our findings of the HPWS–OCB relationship, consolidates the role of the social exchange mechanism in linking HRM systems to OCB. However, this research is different from other studies. First, previous research that asked managers to rate HPWS (Gong et al., 2009, 2010; Snape and Redman, 2010) and asked employees to rate their *perception* about HPWS was applied to all employees in organizations (Shen and Benson, 2014; Zhang et al., 2014). This present study, however, used the employee’s *personal experience* of HPWS to predict OCB. This design is rooted in the idiosyncrasy arguments that employees from the same firm have different experiences of HRM practices (Liu et al., 2013; Wang et al., 2020). It also responds to the call by Wright and Nishii (2006) to examine the impact of an employee’s experience of HR systems on their outcomes. Second, previous HRM-OCB research conducted in Chinese firms employed OCB scales development in Western-country-based studies (Sun et al., 2007; Gong et al., 2009, 2010; Zhang et al., 2014; Newman et al., 2015). We used an OCB scale developed in the Chinese context to reflect the combination of employee traditionality and modernity (Farh et al., 1997).

Secondly, we found that perceived error aversion culture negatively moderated the positive relationship between employee-experienced HPWS and employee OCB. This finding deepens our understanding about the boundary effects of the HRM-OCB relationship by identifying the role of organizational culture. According to social-exchange-based HRM literature, HRM systems can shape the employee-organization relationship, so that employees reciprocate to the organization with OCB through exchanging social resources (Gong et al., 2009, 2010). However, the exchange relationship may be shaped by multiple organizational factors besides HRM systems (Tavares et al., 2016), and organizational culture can be one of these factors. The multiple organizational factors can provide different social resources, and those resources can be aligned with or opposite to each other. That means that the multiple sources of social resources in an organization jointly determine the content of social resources provided to employees for exchanges. It also echoes the idea that employees respond to experienced organizational practices that not only meet their needs, but also are in their favor (Nishii and Wright, 2008; Wang et al., 2020).

Thirdly, we found that perceived error aversion culture and organizational identification jointly moderate the relationship between employee-experienced HPWS and OCB, such that the positive relationship is the strongest when perceived error aversion culture and organizational identification are both low. This is aligned with the idea in the literature that whether or not an employee holds a self-identify or organization identity can shift employee reciprocation of behaviors to a self-focused or organization-focused social exchange relationship (van Knippenberg and Schippers, 2007; Tavares et al., 2016). Our research deepens the understanding about the role of employee identity in intervening in the relationship between HPWS and OCB. Multiple factors in an organization can provide different resources to exch‑ange with employees, and employee identified foci will determine to which organization factors employees are more willing to reciprocate behaviors.

Moreover, our research also challenges the mature idea in HRM-OCB literature that the compatibility between employees’ personalities and organizational values, namely organizational identification can enhance OCB (Fu et al., 2014; Shen et al., 2018; Zulfiqar and Khan, 2021). By drawing on a China-specific management context, we contend that under certain conditions, identification with organizational values may not explain the emergence or enhancement of OCB. The reason is that organizational values can either nurture or inhibit employee OCB performance. Whether or not identifying with organization can cultivate employee OCB depends on the nature of the organizational culture. In this research, although we did not propose the direct effects of error aversion culture on employee OCB, the multiple regression result of Model 2 in Table 3 shows that this culture is negatively and significantly related to employee OCB (β = −0.079, *p* < 0.01). The result is as we expected, that error aversion culture inhibits employee OCB. In such a type of organizational culture, identifying with the organization will inhibit rather than encourage employees to perform OCB, and weaken rather strengthen HRM practices’ effectiveness in cultivating employee OCB.

Finally, our findings can also raise scholarly attention to capturing the joint effect of identification and culture in determining the strength of the HRM-OCB relationship. Findings of our study reveal that an employee’s organizational identification can substantially enhance the moderating effects of perceived error aversion culture. Thus, this once again emphasizes the significance of organizational identification with organizational values in mapping the antecedent effects on employee OCB, or perhaps also on other behaviors.

## Practical Implications

Our research findings have practical implications for managers working in Chinese firms. In spite of the widely recognized usefulness of HPWS in encouraging employees to perform OCB, managers can make more effort to calibrate the implementation of HPWS in the cultural context of their organization. There may be some cultures abating both OCB and HPWS’s effectiveness in cultivating OCB. An error aversion culture is one such example, which still remains in Chinese firms. To facilitate positive effects of HPWS on OCB, firms can make efforts to build an error mastery culture. In such a culture, “it is accepted that error occurrence cannot be eliminated, but at the same time errors are taken seriously and dealt with actively. And there is a focus on detection, correction, communication and learning” (van Dyck, 2009, p. 25).

Further, building up an employee’s mindset of “oneness” or “wholeness” is often deemed to be good for employees to perform OCB. However, caution must be adopted in doing this, since an employee’s identification orientation will direct the employee to exchange with the sources with which they identify. It is advisable for managers to evaluate the content and strength of social resources from both collective-focused and self-focused sources, and adjust their HRM practices (e.g., selection and training) to align individual employees’ cultural preference with that required by the organization.

### Limitations and Suggestions

Our findings should be interpreted in light of the limitations of the study. First, as we used a cross-sectional design, this research cannot examine the causal effects of employee-experienced HPWS and OCB. Although its focus is to explore and examine the boundary effect of HRM-OCB linkage and previous research has used time-lagged data to examine the causality of this relationship, future research may adopt a longitudinal design. Second, this research synthesized multiple theoretical perspectives to explore the boundary conditions of the HRM-OCB relationship. We adopted error aversion culture and organizational identification as moderators in that relationship. Future research can incorporate other theories and further test the role of other variables in intervening in the HRM-OCB linkage. For example, from an institutional perspective, researcher could explore and examine the interventions of macro- or meso-level institutional variables. Third, our research focused on the relationship between individual-level variables. Future research may adopt a multi-level design in investigating the cross-level interactive effects on OCB. Finally, the research draws on a China-specific context: the co-existence of HPWS and an error aversion culture. Such a context is China-specific, but it may be observed in other countries. Future research may replicate the research in other countries and cultures.

## Data Availability Statement

The datasets generated for this study are available on request to the corresponding author.

## Author Contributions

BZ and FC: conceptualization. BZ, PZ, and BS: methodology. BZ and LL: data collection and writing-original draft. PZ and LL: data analysis. BZ: funding acquisition. SZ and XS: project administration. BZ, BS, XS, and YB: writing – review and editing. All authors contributed to the article and approved the submitted version.

## Funding

This research was funded by National Natural Science Foundation of China (NSFC No. 71702116), the Fundamental Research Funds for the Central University (No. buctrc202022).

## Conflict of Interest

The authors declare that the research was conducted in the absence of any commercial or financial relationships that could be construed as a potential conflict of interest.

## Publisher’s Note

All claims expressed in this article are solely those of the authors and do not necessarily represent those of their affiliated organizations, or those of the publisher, the editors and the reviewers. Any product that may be evaluated in this article, or claim that may be made by its manufacturer, is not guaranteed or endorsed by the publisher.

## References

[ref1] Aiken L. S. West S. G. (1991). Multiple regression: testing and interpreting interactions Newbury Park, CA: sage. J. Oper. Res. Soc. 45, 119–120. doi: 10.1057/jors.1994.16

[ref001] Alvesson M. Kärreman D. (2007). Unraveling HRM: Identity, ceremony, and control in a management consulting firm. Organ. Sci. 18, 711–723. doi: 10.1287/orsc.1070.0267

[ref2] Arthur J. B. (1994). Effects of human resource systems on manufacturing performance and turnover. Acad. Manag. J. 37, 670–687. doi: 10.5465/256705

[ref80] Ashforth B. E. Mael F. (1989). Social identity theory and the organization. Acad. Manage. Rev. 14, 20–39. doi: 10.5465/amr.1989.4278999, PMID: 34975677

[ref3] Asplund K. (2020). When profession trumps potential: The moderating role of professional identification in employees' reactions to talent management. Int. J. Hum. Resour. Manag. 31, 539–561. doi: 10.1080/09585192.2019.1570307

[ref4] Baron J. N. Kreps D. M. (2003). Strategic human resources: frameworks for general managers.

[ref5] Bergeron D. M. Shipp A. J. Rosen B. Furst S. A. (2012). Organizational citizenship behavior and career outcomes the cost of being a good citizen. J. Manag. 39, 958–984. doi: 10.1177/0149206311407508

[ref6] Blau G. J. (1986). Job involvement and organizational commitment as interactive predictors of tardiness and absenteeism. J. Manag. 12, 577–584. doi: 10.1177/014920638601200412

[ref7] Brislin R. W. (1970). Back-translation for cross-cultural research. J. Cross-Cult. Psychol. 1, 185–216. doi: 10.1177/135910457000100301

[ref8] Callea A. Urbini F. Chirumbolo A. (2016). The mediating role of organizational identification in the relationship between qualitative job insecurity, OCB and job performance. J. Manag. Dev. 35, 735–746. doi: 10.1108/JMD-10-2015-0143

[ref9] Caniëls M. C. J. Hatak I. (2019). Employee resilience: considering both the social side and the economic side of leader-follower exchanges in conjunction with the dark side of followers' personality. Int. J. Hum. Resour. Manag. 1–32. doi: 10.1080/09585192.2019.1695648

[ref11] Cooke F. L. (2017). Concepts, contexts, and mindsets: putting human resource management research in perspectives. Hum. Resour. Manag. J. 28, 1–13. doi: 10.1111/1748-8583.12163

[ref73] Cropanzano R. Mitchell M. S. (2005). Social exchange theory: an interdisciplinary review. J. Manage. 31, 874–900. doi: 10.1177/0149206305279602

[ref12] Dawson J. F. Richter A. W. (2006). Probing three-way interactions in moderated multiple regression: development and application of a slope difference test. J. Appl. Psychol. 91, 917–926. doi: 10.1037/0021-9010.91.4.917, PMID: 16834514

[ref13] Delery J. E. Doty D. H. (1996). Modes of theorizing in strategic human resource management: tests of universalistic, contingency, and configurational performance predictions. Acad. Manag. J. 39, 802–835. doi: 10.2307/256713

[ref77] Du P. C. Chen Y. (2019). The influence of error aversion culture on turnover intention of new generation employees. East China Econ. Manag. 33, 140–146. doi: 10.19629/j.cnki.34-1014/f.180608003

[ref14] Farh J. L. Earley P. C. Lin S. C. (1997). Impetus for action: a cultural analysis of justice and organizational citizenship behavior in Chinese society. Adm. Sci. Q. 42, 421–444. doi: 10.2307/2393733

[ref15] Farh J. Zhong C. B. Organ D. W. (2004). Organizational citizenship behavior in the People's Republic of China. Organ. Sci. 15, 241–253. doi: 10.1287/orsc.1030.0051

[ref81] Foss N. J. Pedersen T. Fosgaard M. R. Stea D. (2014). Why complementary HRM practices impact performance: the case of rewards, job design, and work climate in a knowledge-sharing context. Hum. Resour. Manage. 54. doi: 10.1002/hrm.21649

[ref17] Fu H. Ye B. H. Law R. (2014). You do well and I do well? The behavioral consequences of corporate social responsibility. Int. J. Hosp. Manag. 40, 62–70. doi: 10.1016/j.ijhm.2014.03.004

[ref79] Gautam T. Dick R. V. Wagner U. (2004). Organizational identification and organizational commitment: Distinct aspects of two related concepts. Asian J. Soc. Psychol. 7, 301–315. doi: 10.1111/j.1467-839X.2004.00150.x

[ref18] Gong Y. Law K. S. Chang S. Xin K. R. (2009). Human resources management and firm performance: the differential role of managerial affective and continuance commitment. J. Appl. Psychol. 94, 263–275. doi: 10.1037/a0013116, PMID: 19186911

[ref19] Gong Y. Song C. Cheung S. Y. (2010). High performance work system and collective OCB: a collective social exchange perspective. Hum. Resour. Manag. J. 20, 119–137. doi: 10.1111/j.1748-8583.2010.00123.x

[ref20] Gouldner A. W. (1960). The norm of reciprocity: a preliminary statement. Am. Sociol. Rev. 25, 161–178. doi: 10.2307/2092623

[ref21] Greenberg D. N. (1995). Blue versus gray: a metaphor constraining sensemaking around a restructuring. Group Org. Manag. 20, 183–209. doi: 10.1177/1059601195202007

[ref22] Halbesleben J. Neveu J. P. Paustian-Underdahl S. C. Westman M. (2014). Getting to the “COR”: understanding the role of resources in conservation of resources theory. J. Manag. 40, 1334–1364. doi: 10.1177/0149206314527130

[ref24] Hernaus T. Černe M. Škerlavaj M. (2021). The interplay between relational job design and cross-training in predicting employee job/task citizenship performance. Hum. Resour. Dev. Q. 34, 625–646. doi: 10.1002/hrdq.21427

[ref25] Hockey G. R. Sauer J. (1996). Cognitive fatigue and complex decision making under prolonged isolation and confinement. Adv. Space Biol. Med. 5, 309–330. doi: 10.1016/S1569-2574(08)60067-2 8814806

[ref82] Hogg M. A. Terry D. J. (2000). Social identity and self-categorization processes in organizational contexts. Acad. Manage. Rev. 25, 121–140. doi: 10.5465/amr.2000.2791606

[ref27] Jackson S. E. Schuler R. S. Jiang K. (2014). An aspirational framework for strategic human resource management. Acad. Manag. Ann. 8, 1–56. doi: 10.5465/19416520.2014.872335

[ref29] Jiang K. Lepak D. P. Hu J. Baer J. C. (2012). How does human resource Management influence organizational outcomes? A meta-analytic investigation of mediating mechanisms. Acad. Manag. J. 55, 1264–1294. doi: 10.5465/amj.2011.0088

[ref30] Kaufman B. E. (2015). Evolution of strategic HRM as seen through two founding books: a 30th anniversary perspective on development of the field. Hum. Resour. Manag. 54, 389–407. doi: 10.1002/hrm.21720

[ref31] Kim S. Wright P. M. Su Z. (2012). Human resource management and firm performance in China: a critical review. Asia Pac. J. Hum. Resour. 48, 58–85. doi: 10.1177/1038411109356496

[ref32] Lepak D. P. Hui L. Chung Y. Harden E. E. (2006). A conceptual review of human resource management systems in strategic human resource Management research. Res. Pers. Hum. Resour. Manag. 25, 217–271. doi: 10.1016/S0742-7301(06)25006-0

[ref34] Liao H. Toya K. Lepak D. P. Hong Y. (2009). Do they see eye to eye? Management and employee perspectives of high-performance work systems and influence processes on service quality. J. Appl. Psychol. 94, 371–391. doi: 10.1037/a0013504, PMID: 19271796

[ref35] Liu J. Lee C. Hui C. Kwan H. K. Wu L. Z. (2013). Idiosyncratic deals and employee outcomes: the mediating roles of social exchange and self-enhancement and the moderating role of individualism. J. Appl. Psychol. 98, 832–840. doi: 10.1037/a0032571, PMID: 23544480

[ref36] Ma L. Liu C. (2019). The research on the mechanism of error orientation to Employees' innovation behavior—the moderating role of misplay culture. Sci. Technol. Prog. Policy 36, 146–152. doi: 10.6049/kjjbydc.L201808449

[ref37] Mael F. Ashforth B. E. (1992). Alumni and their alma mater: A partial test of the reformulated model of organizational identification. J. Organ. Behav. 13, 103–123. doi: 10.1002/job.4030130202

[ref38] Molm L. D. Gretchen P. Nobuyuki T. (2001). The value of exchange. Soc. Forces 80, 159–184. doi: 10.1353/sof.2001.0081

[ref39] Newman A. Nielsen I. Miao Q. (2015). The impact of employee perceptions of organizational corporate social responsibility practices on job performance and organizational citizenship behavior: evidence from the Chinese private sector. Int. J. Hum. Resour. Manag. 26, 1226–1242. doi: 10.1080/09585192.2014.934892

[ref40] Nishii L.H. Wright P.M. (2008). Variability Within Organizations: Implications for Strategic Human Resource Management.

[ref72] Organ D. W. Podsakoff P. M. MacKenzie S. B. (2006). Organizational Citizenship Behavior: Its Nature, Antecedents, and Consequences. Thousand Oaks, CA: Sage.

[ref44] Podsakoff N. P. Whiting S. W. Podsakoff P. M. Blume B. D. (2009). Individual- and organizational-level consequences of organizational citizenship behaviours: a meta-analysis. J. Appl. Psychol. 94, 122–141. doi: 10.1037/a0013079, PMID: 19186900

[ref83] Schein P.A. Schein E. H. (2016). Organizational Culture and Leadership, *5th Edn*. San Francisco, CA: Jossey-Bass Inc Pub.

[ref45] Shen J. Benson J. (2014). When CSR is a social norm: how socially responsible human resource management affects employee work behaviour. J. Manag. 42, 1723–1746. doi: 10.1177/0149206314522300

[ref46] Shen J. Dumont J. Deng X. (2018). Employees’ perceptions of green HRM and non-green employee work outcomes: The social identity and stakeholder perspectives. Group Org. Manag. 43, 594–622. doi: 10.1177/1059601116664610

[ref47] Smidts A. H. Riel C. Pruyn A. (2000). The impact of employee communication and perceived external prestige on organizational identification. Acad. Manag. J. 49, 1051–1062. doi: 10.5465/3069448

[ref48] Snape E. Redman T. (2010). HRM practices, organizational citizenship behaviour, and performance: a multi-level analysis. J. Manag. Stud. 47, 1219–1247. doi: 10.1111/j.1467-6486.2009.00911.x

[ref49] Sun L. Y. Aryee S. Law K. S. (2007). High-performance human resource practices, citizenship behavior, and organizational performance: a relational perspective. Acad. Manag. J. 50, 558–577. doi: 10.2307/20159873

[ref50] Tajfel H. E. (1978). Differentiation Between Social Groups: Studies In The Social Psychology Of Intergroup Relations. London: Academic Press.

[ref51] Takeuchi R. Lepak D. P. Wang H. Takeuchi K. (2007). An empirical examination of the mechanisms mediating between high-performance work systems and the performance of Japanese organizations. J. Appl. Psychol. 92, 1069–1083. doi: 10.1037/0021-9010.92.4.1069, PMID: 17638466

[ref52] Tavares S. M. van Knippenberg D. Dick R. V. (2016). Organizational identification and "currencies of exchange": integrating social identity and social exchange perspectives. J. Appl. Soc. Psychol. 46, 34–45. doi: 10.1111/jasp.12329

[ref53] Tsui A. S. Hui W. Xin K. R. (2010). Organizational culture in China: an analysis of culture dimensions and culture types. Manag. Organ. Rev. 2, 345–376. doi: 10.1111/j.1740-8784.2006.00050.x

[ref54] Tsui A. S. Jone L. P. Porter L. W. Tripoli A. M. (1997). Alternative approaches to the employee-organization relationship: does investment in employees pay off? Acad. Manag. J. 40, 1089–1121. doi: 10.2307/256928

[ref55] Valeau P. J. Paillé P. (2019). The management of professional employees: linking progressive HRM practices, cognitive orientations and organizational citizenship behavior. Int. J. Hum. Resour. Manag. 30, 2705–2731. doi: 10.1080/09585192.2017.1332671

[ref56] van Dyck C. (2009). The tragic 1996 Everest expedition: a tale of error culture. Neth. J. Psychol. 65, 22–34. doi: 10.1007/BF03080124

[ref57] van Dyck C. Frese M. Baer M. Sonnentag S. (2005). Organizational error management culture and its impact on performance: a two-study replication. J. Appl. Psychol. 90, 1228–1240. doi: 10.1037/0021-9010.90.6.1228 16316276

[ref58] van Knippenberg D. Schippers M. C. (2007). Work group diversity. Annu. Rev. Psychol. 58, 515–541. doi: 10.1146/annurev.psych.58.110405.085546 16903805

[ref59] van Knippenberg D. Sleebos E. (2006). Organizational identifications versus organizational commitment: self-definition, social exchange, and job attitudes. J. Organ. Behav. 27, 571–584. doi: 10.1002/job.359

[ref60] Wang Y. K. Kim S. Rafferty A. Sanders K. (2020). Employee perceptions of HR practices: a critical review and future directions. Int. J. Hum. Resour. Manag. 31, 128–173. doi: 10.1080/09585192.2019.1674360

[ref61] Wei L. Q. Liu J. Zhang Y. Chiu R. K. (2008). The role of corporate culture in the process of strategic human resource management: evidence from Chinese enterprises. Hum. Resour. Manag. 47, 777–794. doi: 10.1002/hrm.20244

[ref62] Wikhamn W. Asplund K. Dries N. (2021). Identification with management and the organisation as key mechanisms in explaining employee reactions to talent status. Hum. Resour. Manag. J. 34, 956–976. doi: 10.1111/1748-8583.12335

[ref63] Wright P.M. Nishii L.H. (2006). Strategic HRM and Organizational Behavior: Integrating Multiple Levels of Analysis.

[ref74] Xiao Q. Cooke F. L. (2020). Contextualizing employee perceptions of human resource management: a review of china‐based literature and future directions. Asia Pac. J. Hum. Resour. doi: 10.1111/1744-7941.12259

[ref65] Xiao Z. Tsui A. S. (2007). When brokers may not work: The cultural contingency of social Capital in Chinese High-tech Firms. Adm. Sci. Q. 52, 1–31. doi: 10.2189/asqu.52.1.1

[ref75] Yang K. S. (1993). “Chinese social orientation: An integrative analysis” in Psychotherapy for the Chinese: Selected Papers from the First International Conference. eds. Cheng L. Y. Cheung F. M. C. Chen C. N. (Hong Kong, FL: CUHK Press), 19–56.

[ref66] Zellars K. L. Tepper B. J. (2003). Beyond social exchange: new directions for organizational citizenship behavior theory and research. Res. Pers. Hum. Resour. Manag. 22, 395–424. doi: 10.1016/S0742-7301(03)22009-0

[ref67] Zhang M. Fan D. D. Zhu C. J. (2014). High-performance work systems, corporate social performance and employee outcomes: exploring the missing links. J. Bus. Ethics 120, 423–435. doi: 10.1007/s10551-013-1672-8

[ref68] Zhang Z. Jia M. (2010). Using social exchange theory to predict the effects of high-performance human resource practices on corporate entrepreneurship: evidence from China. Hum. Resour. Manag. 49, 743–765. doi: 10.1002/hrm.20378

[ref70] Zou X. C. Tam K. P. Morris M. Lee S. L. Lau Y. M. Chiu C. Y. (2009). Culture as common sense: perceived consensus versus personal beliefs as mechanisms of cultural influence. Soc. Sci. Electron. Publ. 97, 579–597. doi: 10.1037/a0016399, PMID: 19785480

[ref71] Zulfiqar S. Khan M. S. (2021). Mediating role of organizational citizenship behavior andmoderating role of collectivism and leader–member exchange. Knowl. Process. Manag. doi: 10.1002/kpm.1691

